# 

**Published:** 1889-12

**Authors:** 


### NOTICE.

### The International Medical Congress of 1890.

As organizing committee of the International Medical Congress to be held in Berlin next year, Professors Virchow, Von Bergmann and Waldeyer have issued the following resolutions: The Congress is to be opened on August 4th and closes on the 10th. It is to consist of diplomated physicians and surgeons who have registered themselves as members and have taken tickets of membership. The registration fee is twenty marks, and each member will receive a copy of the Transactions. The purpose of the Congress is purely scientific, and its business will be transacted in Sections. The Committee of Organization will cause the definitive officials to be elected in the first sitting of the Congress —namely: a President, three Vice-Presidents, and an indefinite number of honorary Presidents and Secretaries. In the first sittings of the various Sections a President and a sufficient number of honorary Presidents will be elected, the latter to preside alternately with the former. Owing to the difference of language, a sufficient number of Secretaries will be appointed from among the foreign members. After the Congress, the Transactions will be edited and published by an editing committee appointed by the presiding officials. The general sittings are intended for debates regarding the work and general relations of the Congress, and for addresses and communications of general interest. Such addresses are to be delivered only by members requested to do so by the Committee of Organization. Proposals relating to the work of the Congress must be made to the said Committee before July 1, 1890. The Committee will decide whether they shall be adopted or not. All discourses and communications in the general and in the sectional meetings must be delivered to the Secretaries in writing before the close of the sitting. The Editing Committee will decide whether and in what compass these writings are to be printed in the Transactions. The official languages used at all meetings will be German, English and French. The by-laws and programmes will be printed in all three languages. It is permitted, however, to use another language at the meetings for brief remarks, provided that one of the members present undertakes to communicate the meaning of such remarks in one of the official languages. Introductory discourses in the Sections are to be limited, as a rule, to twenty minutes; in discussion, only ten minutes are allowed to each speaker. Students of medicine, and other persons of both sexes who are not physicians but feel interested in the debates, may be invited by the President, or may receive permission to attend the sittings.

The Imperial German Government has taken upon itself the duty of providing part of the funds requisite for the coming International Medical Congress, to be held in Berlin, August, 1890, to the extent of 80,000 marks. It is expected that the Prussian State Treasury will be still more liberal.

### EDITORIAL.

### Close of the Volume.

With this number of the REGISTER the forty-third volume is completed. The first year of its existence it was published by the Mississippi Dental Society and edited by a committee consisting of Drs. James Taylor, of Cincinnati; S. P. Wullihen, of Wheeling, Va., and B. B. Brown, of St. Louis.

At the beginning of the second volume Dr. J. Taylor became the sole editor, publisher and proprietor. It continued in his hands 'till the completion of the ninth volume, at which time it became the property of J. Taft and Geo. Watt and was edited and published by them till volume 25. To 1859 it was a quarterly, at that time it was converted into a monthly, being the first monthly dental journal in this country, except the first volume of the *American Journal of Dental Science*, 1839-40. Prior to 1865 its title was "The Dental Register of the West;" at this date the latter part of the title was dropped, and it has remained up to the present time as it now stands. It has been for the last eighteen years solely in charge of the present editor. His editorial work on this publication has continued without interruption for thirty-four years.

During this time a growth and development in dental science and art has taken place that the most sanguine did not venture to predict. This has been effected by the combined influence of many factors and forces, to no single person or circumstance can a very large per cent. of this result be attributed. It has been by accretion from a thousand sources and through many avenues.

What the future may bring forth can not now be known, but if the past may be regarded as prophetic of the future, we are warranted in the expectation that progress equal to, if not greater, will be made in the future than in the past. Just what this will be it is needless to speculate. There is work enough and room enough for all the agencies that are now in operation and there is no occasion for clashing, antagonism nor vain boasting.

Better would it be if all our instrumentalities could be helpful to each other, and in the fullest sense co-operative. It is gratifying to know that this is, to some extent, at least, being realized, but there is yet much to be done, and it is to be hoped that the incoming year will be more prolific in this respect than the one just closing.

The Register will endeavor to do its part in the coming year as in the past.

ESTABLISHED 1845.

### Ohio College of Dental Surgery.

Department of Dentistry—University of Cincinnati.

### SESSION 1888-89.

### FACULTY.

J. S. CASSIDY, M.D., D.D.S., Professor of Chemistry and Materia Medica.
H. A. SMITH, D.D.S., DEAN OF THE FACULTY, Professor of Operative Dentistry and Dental Pathology.
C. M. WRIGHT, D.D.S., Professor of Physiology and General Pathology.
WM. KNIGHT, M.D., D.D.S., Professor of Anatomy and Oral Surgery.
GRANT MOLLYNEAUX, D.D.S., Professor of Mechanical Dentistry and Metallurgy.

### DEMONSTRATORS.

B. C. HINKLEY, D.D.S., J. E. BARRICKLOW, D.D.S., } Demonstrators of Operative Dentistry.
G. S. JUNKERMAN, M.D., D.D.S., Demonstrator of Analytical Chemistry.
H. C. MATLACK, D.D.S., Demonstrator of Anatomy.
C. NEIDHAMER, Demonstrator of Mechanical Dentistry.

The Forty-Third Annual Winter Session begins Tuesday, October 2, 1888, and closes March 1, 1889.

The Annual Spring Session begins about April 1, 1889, and continues eight weeks.

The requirements for admission and graduation are those adopted by the National Association of Dental Faculties.

### FEES.

| | |
|---|---|
| Marticulation Fee (but once) | $ 5.00 |
| Professors' Tickets | 75.00 |
| Dissecting Ticket, including Material | 10.00 |
| Analytical Chemistry | 10.00 |
| Diploma Fee | 25.00 |
| Spring Course Ticket | 20.00 |

For further information and announcements, address

**H. A. SMITH, Dean, 128 Garfield Place, Cincinnati, Ohio.**

ESTABLISHED 1845.

### Ohio College of Dental Surgery.

Department of Dentistry—University of Cincinnati.

### SESSION 1888-89.

### FACULTY.

J. S. CASSIDY, M.D., D.D.S., Professor of Chemistry and Materia Medica.

H. A. SMITH, D.D.S., Dean of the Faculty, Professor of Operative Dentistry and Dental Pathology.

C. M. WRIGHT, D.D.S., Professor of Physiology and General Pathology.

WM. KNIGHT, M.D., D.D.S., Professor of Anatomy and Oral Surgery.

GRANT MOLLYNEAUX, D.D.S., Professor of Mechanical Dentistry and Metallurgy.

### DEMONSTRATORS.

B. C. HINKLEY, D.D.S.,<br>J. E. BARRICKLOW, D.D.S., } Demonstrators of Operative Dentistry.

G. S. JUNKERMAN, M.D., D.D.S., Demonstrator of Analytical Chemistry.

H. C. MATLACK, D.D.S., Demonstrator of Anatomy.

C. NEIDHAMER, Demonstrator of Mechanical Dentistry.

The Forty-Third Annual Winter Session begins Tuesday, October 2, 1888, and closes March 1, 1889.

The Annual Spring Session begins about April 1, 1889, and continues eight weeks.

The requirements for admission and graduation are those adopted by the National Association of Dental Faculties.

### FEES.

| | |
|---|---|
| Marticulation Fee (but once) | $ 5.00 |
| Professors' Tickets | 75.00 |
| Dissecting Ticket, including Material | 10.00 |
| Analytical Chemistry | 10.00 |
| Diploma Fee | 25.00 |
| Spring Course Ticket | 20.00 |

For further information and announcements, address

**H. A. SMITH, Dean, 128 Garfield Place, Cincinnati, Ohio.**

ESTABLISHED 1845.

### Ohio College of Dental Surgery.

Department of Dentistry—University of Cincinnati.

### SESSION 1888-89.

### FACULTY.

J. S. CASSIDY, M.D., D.D.S., Professor of Chemistry and Materia Medica.
H. A. SMITH, D.D.S., DEAN OF THE FACULTY, Professor of Operative Dentistry and Dental Pathology.
C. M. WRIGHT, D.D.S., Professor of Physiology and General Pathology.
WM. KNIGHT, M.D., D.D.S., Professor of Anatomy and Oral Surgery.
GRANT MOLLYNEAUX, D.D.S., Professor of Mechanical Dentistry and Metallurgy.

### DEMONSTRATORS.

B. C. HINKLEY, D.D.S.,
J. E. BARRICKLOW, D.D.S., } Demonstrators of Operative Dentistry.
G. S. JUNKERMAN, M.D., D.D.S., Demonstrator of Analytical Chemistry.
H. C. MATLACK, D.D.S., Demonstrator of Anatomy.
C. NEIDHAMER, Demonstrator of Mechanical Dentistry.

The Forty-Third Annual Winter Session begins Tuesday, October 2, 1888, and closes March 1, 1889.

The Annual Spring Session begins about April 1, 1889, and continues eight weeks.

The requirements for admission and graduation are those adopted by the National Association of Dental Faculties.

### FEES.

| | |
|---|---|
| Marticulation Fee (but once) | $ 5.00 |
| Professors' Tickets | 75.00 |
| Dissecting Ticket, including Material | 10.00 |
| Analytical Chemistry | 10.00 |
| Diploma Fee | 25.00 |
| Spring Course Ticket | 20.00 |

For further information and announcements, address

H. A. SMITH, Dean, 128 Garfield Place, Cincinnati, Ohio.

### DIRECTORY OF DENTAL SOCIETIES.

*American Dental Association*—Meets on the first Tuesday of August, 1889 at Saratoga. Pres. C. R. Butler, Cleveland, O.; Rec. Sec. Geo. H. Cushing, Chicago.

*Southern Dental Association*—Meets in Galveston, Texas. President J. Y. Crawford, Nashville, Tenn., Secretary M. C. Marshall, Little Rock, Ark.

### STATE DENTAL SOCIETIES.

*Alabama Dental Association*—Meets annually. Next meeting at Birmingham, on the second Tuesday of April, 1889. Pres. J. C. Wilkerson, Selma; Sec. R. Y. Jones, Florence.

*California State Dental Association*—Third Tuesday in July, 1889. Pres. W. De Crow, San Josie; Rec. Sec. W. A. Knowles, San Francisco; Cor. Sec. W. Z. King, San Francisco. Next Meeting in San Francisco.

*Colorado State Dental Society*—Pres. J. W. Grannis, Sec. H. P. Kellog, Denver; Next meeting will be held in Denver, June 4th, 1889.

*Connecticut State Dental Society*—Pres. E. A. Stebbins, Shelburne Falls; Rec. Sec. Geo. A. Maxfield, Holyoke. Annual meeting Oct. 27th and 28th, 1889, at Hartford.

*Dental Society of the State of Maryland and District of Columbia*—Meets annually. Next meeting at Washington City, on the second Tuesday in October, 1889. Pres. B. F. Coy, of Baltimore: Sec. M. W. Foster, M. D.

*Georgia State Dental Society*—Meets second Tuesday in May, 1889, at Tybee; Pres. S. A. White: Rec. Sec. H. H. Johnson; Cor. Sec. L. D. Carpenter, Atlanta.

*Illinois State Dental Society*—Meets annually. Pres. Geo. H. Cushing, Chicago; Sec. G. Newkirk, Chicago. Next place of meeting, Quincy, second Tuesday in May, 1889.

*Indiana State Dental Society*—Meets next in Indianapolis on the last Tuesday of June 1898. Pres. J. B. Morrison, Indianapolis; Sec. Robert W. Van Valzah, Terre Haute.

*Iowa State Dental Society*—Meets annually. Next meeting in Dubuque, on the first Tuesday in May, 1890. Pres. F. M. Shriver, Glenwood; Rec. Sec. G. W. Miller, Des Moines.

*Kentucky State Dental Association*—Meets annually, first Tuesday in June, 1889. Next meeting in Louisville. Pres. J. M. Baldwin, Sec. C. E. Dunn. Louisville.

*Kansas State Dental Association*—Pres. W. M. Shirley, Hiawatha; Sec. C. B. Gunn, Leavenworth. Next meeting will be held at Topeka, April 30th, 1889.

*Maine Dental Society*—Meets in August, at Thomaston. Pres. E. J. Robert, Augusta, Sec. C. E. Hussey, Beddeford.

*Maryland State Dental Association*—Meets annually in February. Pres. Wm. A. Mills; Rec. Sec. Chas. F. Dingler; Cor. Sec. S. C. Pennington

*Massachusetts Dental Society*—Meets Semi-Annually in Boston, June 5th, 6th and 7th, 1889. Pres. G. A. Gerry, Boston; Rec. Sec. E. O. Kinsman, Boston.

*Michigan State Dental Association*—Meets annually. Next meeting at Grand Rapids, Tuesday, June 7th, 1889. Pres. C. S. Case, Jackson; Sec. Wm. Cleland, Detroit; Treas. H. K. Lathrop, Detroit.

*Mississippi State Dental Association*—Will meet third Tuesday in May, 1889, at Vicksburg. Pres. W. H. Marshall, Oxford; Sec. A. H. Hilzim, Jackson.

*Minnesota State Society*—Pres. L. E. Weeks, Minneapolis; Sec. D. F. Mac Graw, Mankato. Meets at Deluth, 3rd Tuesday in July, for four days.

*Missouri Dental Association*—Meets annually on July 10th, 1889. Next meeting at Pertel Springs. Pres. Wm. N. Morrison, St. Louis; Sec. John G. Harper, St. Louis.

*Nebraska State Dental Society*—Meets annually. Next meeting third Tuesday in May 1889, at Beatrice; Pres. J. J. Willey, Wahoo; Sec. C. R. Tefft, Lincoln.

*New Jersey State Dental Society*—Meets annually. Next meeting at Asbury Park, on the third Wednesday in July, 1889. Pres. A. Hull, New Brunswick; Sec. C. A. Meeker, Newark.

*New Hampshire Dental Society*—Meets in Concord on the third Tuesday of June, 1889. Pres. C. H. Maynard, Peterboro; Sec. E. B. Davis, Concord.

*North Carolina State Dental Society*—Meets annually. Next meeting at Ashville; fourth Tuesday in July, 1889. Pres. V. E. Turner, Raleigh; Sec. H. C. Hearing, Concord.

*Ohio State Dental Society*—Meets annually. Next meeting at Cleveland, last Tuesday of October, 1889. Pres. C. M. Wright, Cincinnati; Sec. J. R. Callahan, Hillsboro.

*Pennsylvania State Dental Society*—Meets second Tuesday of September, 1889, at Philadelphia; Pres. W. H. Roop Philadelphia; Rec. and Cor. Sec. Th. F. Chupein, Philadelphia.

*Rhode Island Dental Association*—Pres. A. W. Buckland, Woonsocket; Sec. W. P. Church, Providence. Meets quarterly, first Tuesday in July, October, January, and April. Annual Meeting first Tuesday in July, 1889.

*South Carolina State Dental Association*—Meets annually. Pres. L. S. Wolfe, Orangburg; Cor. Sec. E. C. Ridgell, Batesburg; Rec. Sec. R. A. Smith, Charleston. Next meeting at Greenville, third Tuesday in July, 1889.

*Texas State Dental Association*—Meets in Buton, May 1st, 1889. Pres. W. J. Barton, Paris; Sec. G. M. Patten, Huntsville.

*Tennessee Dental Association*—Meets Annually Next meeting at Jackson, first Tuesday in July 1889. Pres. A. Gordon White, Springfield; Rec. Sec. J. D. Hanston, Lewisburg; Cor. Sec. Gordon White, Nashville.

*The Dental Society of the State of New York*—Meets annually on the second Wednesday in May. Next session at Albany, May 8, 1889. Pres. J. Edw. Line, Rochester; Rec. Sec. M. S. Jewell, Richfield Spa.; Cor. Sec. G. L. Curtis, Syracuse.

*Vermont State Dental Society*—Meets annually. Next meeting at Bellow's Falls, March 19, 1889. Pres. W. H. Spence, Poultney; Sec. Thos. Maund, Rutland.

*Virginia State Dental Association*—Meets in Charlottsville, Tuesday, August 19, 1888. Pres. J. Hall Moore; Sec. L. M. Cowarden, Richmond.

*Wisconsin State Dental Society*—Meets annually. Next meeting at Milwaukee, July 20, 1889. Pres. B. G. Marklein, Milwaukee; Sec. C. A. Southwell, Appleton, Wis.

### LOCAL SOCIETIES.

*American Academy of Dental Science*—Meets annually in Boston, Mass., on the first Wednesday in October, 1889. Pres. J. H. Batchelder; Rec. Sec. E. E. Hopkins; Cor. Sec. E. B. Hitchcock, Boston.

*Brooklyn Dental Society and Second District Society United*—Meets quarterly—Pres. T. H. Holly; Rec. Sec. A. N. Russell, Brooklyn.

*Central Dental Association of Northern New Jersey*—Meets annually in February. Pres. Geo. E. Adams, South Orange; Sec. J. Allen Osmun, Newark.

*Central Illinois Dental Society*—Meets annually. Next meeting at Lincoln, Oct. Oct. 9 and 10, 1889; Pres. J. M. Fishburn, El Paso; Sec. W. A. Johnston, Peoria.

*Chicago Dental Society*—Meets monthly. Pres. F. H. Gardiner; Sec. J. G. Reid.

*Chicago Dental Club*—Meets on the fourth Monday of each month. Pres. A. B. Freeman; Sec. C. Stoddard Smith.

*Cleveland Dental Society*.—Meets the first Monday of each month. Pres. Ira Brown; Sec. Jere E. Robinson.

*Connecticut Valley Dental Society*—Meets 27 and 28 of Oct. 1889, at Savin Rock Conn. Pres. C. T. Stockwell, Springfield, Mass. Sec. A. M. Ross, Chicopee.

*Detroit Dental Society*—Meets monthly, Pres. E. C. Moore, Sec. Wm. Cleland.

*Eighth District Dental Society, N. Y.*—Meets semi-annually. Next meeting in Syracuse, Oct. 24-26, 1889. Pres. T. E. Howard; Sec. S. A. Freeman, Buffalo.

*East Tennessee Dental Association*—Meets annually. Next meeting will be held at Chattanooga, Tenn., on the first Tuesday of Aug., 1889. Pres. R. A. B. Noyers, Sec. S. N. Prothro.

*Mad River Dental Society*—Meets annually. Next meeting at Dayton, Tuesday, May 19, 1889. Pres. C. Bradley, Dayton; Sec. and Treas. L. C. Adams, Dayton,

*Mississippi Valley Dental Society*—Meets annually at Cincinnati, on first Wednesday in March, 1889. Pres. .................................. Sec. .............. Cincinnati.

*New England Dental Society*—Meets annually. Time and place of next meeting in Boston, November 15th, 1889. Pres., A. M. Dudley, Salem, Mass.; Sec. A. H. Gilson, Boston, Mass.

*Northern Illinois Dental Society*—Meets annually. Next meeting at Freeport, October —, 1889. Pres. J. H. Cormany, Mt. Carroll. Sec. T. W. Beckwith.

*Northern Ohio Dental Association*—Meets annually. Next meeting at Cleveland, O. on the second Tuesday in May, 1889. Pres. H. F. Harvey, Cleveland, O.; Rec. Sec. W. H. Whitsler, Youngstown; Cor. Sec. S. B. Dewey, Cleveland, O.

*Northwestern Dental Association* (embraces portions of Dakota, Minnesota and Manitoba) meets annually. Next meeting at Detroit, Dak. on the fourth Tuesday in July, 1889. Pres. J. W. Cloes, Jamestown, Dak. Sec. S. J. Hill, Fargo, Dak.

*Odontological Society of Pennsylvania*—Meets on the first Saturday evening of each month, at 8 o'clock p. m., in the offices of the members; Pres. E. T. Darby, Rec. Sec. Ambler Tees.

*Odontological Society of New York City*—Meets the third Tuesday of each month. Pres. Wm. Jarvie; Rec. Sec. E. T. Payne.

*Odontological Society of Cincinnati*—Meets monthly. Pres. C. M, Wright; Sec. W. M. Williams.

*Ohio Valley Dental Society.*—Meets quarterly. Next meeting first Monday in July, 1889, at Stuebenville, O. Pres. N. H. Harrison, Cadiz; Sec'y F. S. Maxwell, Stuebenville, O.

*Pennsylvania Association of Dental Surgeons*—Meets second Tuesday of each month; Pres. H. E. Roberts; Rec. Se . Theo. F. Chupein.

*Pittsburg Dental Society*—Meets the last Tuesday Evening of each month. Pres. J. B. Williams, Homestead; Sec. N. L. Reinecke.

*Fifth District Dental Society of N. Y.*—Meets semi-annually. Next meeting in Syracuse, October, 24-26. Pres. R. F. Jones, Utica; Sec. T. B. Mason, Phoenix.

*San Francisco Dental Society*—Meets the second Monday evening of each month. Pres. W. A. Knowles; Sec. Chas. Morffew; Treas. J. J. Birge.

*Sixth District Dental Society of N. Y.*—Meets semi-annually. Next meeting at Syracuse, Oct. 24-26, 1889. Pres. E. D. Downs, Owego; Sec. Myron D. Jewell, Richfield Springs.

*St. Louis Dental Society*—Meets first Tuesday in each month. Pres. A. J. Prosser; Cor. Sec. Wm. Conrad; Rec. Sec. Jessie E. Grasheider.

*Washington Dental Society*—Meets second Tuesday of each month. Pres. R. Finley Hunt; Rec. Sec. H. M. Schooly.

*First District Dental Society of the State of New York*—Meets annually. Pres. A. I. Northrop; Sec. J. E. Dexter, New York.

*Washington City Dental Society*—Pres. M. B. Noble; Sec. Wm. Donally. Meets monthly.

*The Western Illinois District Dental Association*—Meets at Kewanee, October 16, Pres. F. Christianer, Abington; Sec. R. W. Bailey, Macomb.

---

### EXCHANGES

St. Louis, Medical and Surgical Journal.

The Pacific Medical and Surgical Journal, San Francisco.

Cincinnati Lancet and Clinic.

Dental Cosmos, Philadelphia.

Chicago Medical Examiner.

The Detroit Review of Medicine and Pharmacy.

American Journal of Dental Science, Baltimore.

Archives of Dentistry.

L'art Dentaire, Paris.

Le Progres Dentare, Paris.

Deutsche Monatsschrift fuer Zahnheilkunde Leipzig.—Arthur Felix.

Ohio State Journal of Dental Science, Xenia, Ohio

Independent Practitioner, N. Y.

The Dental Record, London.

The Journal of the British Dental Association, London.

The Southern Dental Journal.

Items of Interest, Philadelphia.

The Dental Practitioner.—The Cincinnati "Medical" News.

The British Journal of Dental Science, London, Eng.

The Dental Advertiser.

The Messenger of Dentistry, Russian.

The Medical World.

The American Pharmacist.

The Nashville Journal of Medicine and Surgery.

Medical and Surgical Reporter, Philadelphia, Pa.

Dental Headlight.

Cincinnati Medical and Dental Journal.

The New York Medical and Surgical Journal.

The Dental Review.

The Western Dental Journal.

ESTABLISHED 1845.

### Ohio College of Dental Surgery.

Department of Dentistry—University of Cincinnati.

### SESSION 1888-89.

### FACULTY.

J. S. CASSIDY, M.D., D.D.S., Professor of Chemistry and Materia Medica.

H. A. SMITH, D.D.S., DEAN OF THE FACULTY, Professor of Operative Dentistry and Dental Pathology.

C. M. WRIGHT, D.D.S., Professor of Physiology and General Pathology.

WM. KNIGHT, M.D., D.D.S., Professor of Anatomy and Oral Surgery.

GRANT MOLLYNEAUX, D.D.S., Professor of Mechanical Dentistry and Metallurgy.

### DEMONSTRATORS.

B. C. HINKLEY, D.D.S., } Demonstrators of Operative Dentistry.

J. E. BARRICKLOW, D.D.S., }

G. S. JUNKERMAN, M.D., D.D.S., Demonstrator of Analytical Chemistry.

H. C. MATLACK, D.D.S., Demonstrator of Anatomy.

C. NEIDHAMER, Demonstrator of Mechanical Dentistry.

The Forty-Third Annual Winter Session begins Tuesday, October 2, 1888, and closes March 1, 1889.

The Annual Spring Session begins about April 1, 1889, and continues eight weeks.

The requirements for admission and graduation are those adopted by the National Association of Dental Faculties.

### FEES.

| | |
|---|---|
| Marticulation Fee (but once) | $ 5.00 |
| Professors' Tickets | 75.00 |
| Dissecting Ticket, including Material | 10.00 |
| Analytical Chemistry | 10.00 |
| Diploma Fee | 25.00 |
| Spring Course Ticket | 20.00 |

For further information and announcements, address

H. A. SMITH, Dean, 128 Garfield Place, Cincinnati, Ohio.

### DIRECTORY OF DENTAL SOCIETIES.

*American Dental Association*—Meets on the first Tuesday of August, 1889 at Saratoga. Pres. C. R. Butler, Cleveland, O.; Rec. Sec. Geo. H. Cushing, Chicago.
*Southern Dental Association*—Meets in Galveston, Texas. President J. Y. Crawford, Nashville, Tenn., Secretary M. C. Marshall, Little Rock, Ark.

### STATE DENTAL SOCIETIES.

*Alabama Dental Association*—Meets annually. Next meeting at Birmingham, on the second Tuesday of April, 1889. Pres. J. C. Wilkerson, Selma; Sec. R. Y. Jones, Florence.

*California State Dental Association*—Third Tuesday in July, 1889. Pres. W. De Crow, San Josie; Rec. Sec. W. A. Knowles, San Francisco; Cor. Sec. W. Z. King, San Francisco. Next Meeting in San Francisco.

*Colorado State Dental Society*—Pres. J. W. Grannis, Sec. H. P. Kellog, Denver; Next meeting will be held in Denver, June 4th, 1889.

*Connecticut State Dental Society*—Pres. E. A. Stebbins, Shelburne Falls; Rec. Sec. Geo. A. Maxfield, Holyoke. Annual meeting Oct. 27th and 28th, 1889, at Hartford.

*Dental Society of the State of Maryland and District of Columbia*—Meets annually. Next meeting at Washington City, on the second Tuesday in October, 1889. Pres. B. F. Coy. of Baltimore: Sec. M. W. Foster, M. D.

*Georgia State Dental Society*—Meets second Tuesday in May, 1889, at Tybee; Pres. S. A. White: Rec. Sec. H. H. Johnson; Cor. Sec. L. D. Carpenter, Atlanta.

*Illinois State Dental Society*—Meets annually. Pres. Geo. H. Cushing, Chicago; Sec. G. Newkirk, Chicago. Next place of meeting, Quincy, second Tuesday in May, 1889.

*Indiana State Dental Society*—Meets next in Indianapolis on the last Tuesday of June 1898. Pres. J. B. Morrison, Indianapolis; Sec. Robert W. Van Valzah, Terre Haute.

*Iowa State Dental Society*—Meets annually. Next meeting in Dubuque, on the first Tuesday in May, 1890. Pres. F. M. Shriver, Glenwood; Rec. Sec. G. W. Miller, Des Moines.

*Kentucky State Dental Association*—Meets annually, first Tuesday in June, 1889. Next meeting in Louisville. Pres. J. M. Baldwin, Sec. C. E. Dunn, Louisville.

*Kansas State Dental Association*—Pres. W. M. Shirley, Hiawatha; Sec. C. B. Gunn, Leavenworth. Next meeting will be held at Topeka, April 30th, 1889.

*Maine Dental Society*—Meets in August, at Thomaston. Pres. E. J. Robert, Augusta, Sec. C. E. Hussey, Beddeford.

*Maryland State Dental Association*—Meets annually in February. Pres. Wm. A. Mills; Rec. Sec. Chas. F. Dingler; Cor. Sec. S. C. Pennington

*Massachusetts Dental Society*—Meets Semi-Annually in Boston, June 5th, 6th and 7th, 1889. Pres. G. A. Gerry, Boston; Rec. Sec. E. O. Kinsman, Boston.

*Michigan State Dental Association*—Meets annually. Next meeting at Jackson, Tuesday, June 7th, 1889. Pres. C. S. Case, Jackson; Sec. Wm. Cleland, Detroit; Treas. H. K. Lathrop, Detroit.

*Mississippi State Dental Association*—Will meet third Tuesday in May, 1889, at Vicksburg. Pres. W. H. Marshall, Oxford; Sec. A. H. Hilzim, Jackson.

*Minnesota State Society*—Pres. L. E. Weeks, Minneapolis; Sec. D. F. Mac Graw, Mankato. Meets at Deluth, 3rd Tuesday in July, for four days.

*Missouri Dental Association*—Meets annually on July 10th, 1889. Next meeting at Pertel Springs. Pres. Wm. N. Morrison, St. Louis; Sec. John G. Harper, St. Louis.

*Nebraska State Dental Society*—Meets annually. Next meeting third Tuesday in May 1889, at Beatrice; Pres. J. J. Willey, Wahoo; Sec. C. R. Tefft, Lincoln.

*New Jersey State Dental Society*—Meets annually. Next meeting at Asbury Park, on the third Wednesday in July, 1889. Pres. A. Hull, New Brunswick; Sec. C. A. Meeker, Newark.

*New Hampshire Dental Society*—Meets in Concord on the third Tuesday of June, 1889. Pres. C. H. Maynard, Peterboro; Sec. E. B. Davis, Concord.

*North Carolina State Dental Society*—Meets annually. Next meeting at Ashville; fourth Tuesday in July, 1889. Pres. V. E. Turner, Raleigh; Sec. H. C. Hearing, Concord.

*Ohio State Dental Society*—Meets annually. Next meeting at Cleveland, last Tuesday of October, 1889. Pres. C. M. Wright, Cincinnati; Sec. J. R. Callahan, Hillsboro.

*P.nnsylvania State Dental Society*—Meets second Tuesday of September, 1889, at P[illegible]iladelphia; Pres. W. H. Roop Philadelphia; Rec. and Cor. Sec. Th. F. Chupein, Philadelphia.

*Rhode Island Dental Association*—Pres. A. W. Buckland, Woonsocket; Sec. W. P. Church, Providence. Meets quarterly, first Tuesday in July, October, January, and April. Annual Meeting first Tuesday in July, 1889.

*South Carolina State Dental Association*—Meets annually. Pres. L. S. Wolfe, Orangburg; Cor. Sec. E. C. Ridgell, Batesburg; Rec. Sec. R. A. Smith, Charleston. Next meeting at Greenville, third Tuesday in July, 1889.

*Texas State Dental Association*—Meets in Buton, May 1st, 1889. Pres. W. J. Barton, Paris; Sec. G. M. Patten, Huntsville.

*Tennessee Dental Association*—Meets Annually. Next meeting at Jackson, first Tuesday in July 1889. Pres. A. Gordon White, Springfield; Rec. Sec. J. D. Hanston, Lewisburg; Cor. Sec. Gordon White, Nashville.

*The Dental Society of the State of New York*—Meets annually on the second Wednesday in May. Next session at Albany, May 8, 1889. Pres. J. Edw. Line, Rochester; Rec. Sec. M. S. Jewell, Richfield Spa.; Cor. Sec. G. L. Curtis, Syracuse.

*Vermont State Dental Society*—Meets annually. Next meeting at Bellow's Falls, March 19, 1889. Pres. W. H. Spence, Poultney; Sec. Thos. Maund, Rutland.

*Virginia State Dental Association*—Meets in Charlottsville, Tuesday, August 19, 1888. Pres. J. Hall Moore; Sec. L. M. Cowarden, Richmond.

*Wisconsin State Dental Society*—Meets annually. Next meeting at Milwaukee, July 20, 1889 Pres. B. G. Marklein, Milwaukee; Sec. C. A. Southwell, Appleton, Wis.

### LOCAL SOCIETIES.

*American Academy of Dental Science*—Meets annually in Boston, Mass., on the first Wednesday in October, 1889. Pres. J. H. Batchelder; Rec. Sec. E. E. Hopkins; Cor. Sec. E. B. Hitchcock, Boston.

*Brooklyn Dental Society and Second District Society United*—Meets quarterly—Pres. T. H. Holly; Rec. Sec. A. N. Russell, Brooklyn.

*Central Dental Association of Northern New Jersey*—Meets annually in February. Pres. Geo. E. Adams, South Orange; Sec. J. Allen Osmun, Newark.

*Central Illinois Dental Society*—Meets annually. Next meeting at Lincoln, Oct. Oct. 9 and 10, 1889; Pres. J. M. Fishburn, El Paso; Sec. W. A. Johnston, Peoria.

*Chicago Dental Society*—Meets monthly. Pres. F. H. Gardiner; Sec. J. G. Reid.

*Chicago Dental Club*—Meets on the fourth Monday of each month. Pres. A. B. Freeman; Sec. C. Stoddard Smith.

*Cleveland Dental Society.*—Meets the first Monday of each month. Pres. Ira Brown; Sec. Jere E. Robinson.

*Connecticut Valley Dental Society*—Meets 27 and 28 of Oct. 1889, at Savin Rock Conn. Pres. C. T. Stockwell, Springfield, Mass. Sec. A. M. Ross, Chicopee.

*Detroit Dental Society*—Meets monthly, Pres. E. C. Moore, Sec. Wm. Cleland.

*Eighth District Dental Society, N. Y.*—Meets semi-annually. Next meeting in Syracuse, Oct. 24-26, 1889. Pres. T. E. Howard; Sec. S. A. Freeman, Buffalo.

*East Tennessee Dental Association*—Meets annually. Next meeting will be held at Chattanooga, Tenn., on the first Tuesday of Aug., 1889. Pres. R. A. B. Noyers, Sec. S. N. Prothro.

*Mad River Dental Society*—Meets annually. Next meeting at Dayton, Tuesday, May 19, 1889. Pres. C. Bradley, Dayton; Sec. and Treas. L. C. Adams, Dayton,

*Mississippi Valley Dental Society*—Meets annually at Cincinnati, on first Wednesday in March, 1889. Pres. ..............................Sec. .............. Cincinnati.

*New England Dental Society*—Meets annually. Time and place of next meeting in Boston, November 15th, 1889. Pres., A. M. Dudley, Salem, Mass.; Sec. A. H. Gilson, Boston, Mass.

*Northern Illinois Dental Society*—Meets annually. Next meeting at Freeport, October —, 1889. Pres. J. H. Cormany, Mt. Carroll. Sec. T. W. Beckwith.

*Northern Ohio Dental Association*—Meets annually. Next meeting at Cleveland, O. on the second Tuesday in May, 1889. Pres. H. F. Harvey, Cleveland, O.; Rec. Sec. W. H. Whitsler, Youngstown; Cor. Sec. S. B. Dewey, Cleveland, O.

*Northwestern Dental Association* (embraces portions of Dakota, Minnesota and Manitoba) meets annually. Next meeting at Detroit, Dak. on the fourth Tuesday in July, 1889. Pres. J. W. Cloes, Jamestown, Dak. Sec. S. J. Hill, Fargo, Dak.

*Odontological Society of Pennsylvania*—Meets on the first Saturday evening of each month, at 8 o'clock p. m., in the offices of the members; Pres. E. T. Darby, Rec. Sec. Ambler Tees.

*Odontological Society of New York City*—Meets the third Tuesday of each month. Pres. Wm. Jarvie; Rec. Sec. E. T. Payne.

*Odontological Society of Cincinnati*—Meets monthly. Pres. C. M, Wright; Sec. W. M. Williams.

*Ohio Valley Dental Society.*—Meets quarterly. Next meeting first Monday in July, 1889, at Stuebenville, O. Pres. N. H. Harrison, Cadiz; Sec'y F. S. Maxwell, Stuebenville, O.

*Pennsylvania Association of Dental Surgeons*—Meets second Tuesday of each month; Pres. H. E. Roberts; Rec. Sec. Theo. F. Chupein.

*Pittsburg Dental Society*—Meets the last Tuesday Evening of each month. Pres. J. B. Williams, Homestead; Sec. N. L. Reinecke.

*Fifth District Dental Society of N. Y.*—Meets semi-annually. Next meeting in Syracuse, October, 24-26. Pres. R. F. Jones, Utica; Sec. T. B. Mason, Phoenix.

*San Francisco Dental Society*—Meets the second Monday evening of each month. Pres. W. A. Knowles; Sec. Chas. Morffew; Treas. J. J. Birge.

*Sixth District Dental Society of N. Y.*—Meets semi-annually. Next meeting at Syracuse, Oct. 24-26, 1889. Pres. E. D. Downs, Owego; Sec. Myron D. Jewell, Richfield Springs.

*St. Louis Dental Society*—Meets first and third Tuesday in each month. Pres. A. J. Prosser; Cor. Sec. Wm. Conrad; Rec. Sec. Jessie E. Grosheider.

*Washington Dental Society*—Meets second Tuesday of each month. Pres. R. Finley Hunt; Rec. Sec. H. M. Schooly.

*First District Dental Society of the State of New York*—Meets annually. Pres. A. L. Northrop; Sec. J. E. Dexter, New York.

*Washington City Dental Society*—Pres. M. B. Noble; Sec. Wm. Donally. Meets monthly.

*The Western Illinois District Dental Association*—Meets at Kewanee, October 16, Pres. F. Christianer, Abington; Sec. R. W. Bailey, Macomb.

---

### EXCHANGES

St. Louis, Medical and Surgical Journal.
The Pacific Medical and Surgical Journal, San Francisco.
Cincinnati Lancet and Clinic.
Dental Cosmos, Philadelphia.
Chicago Medical Examiner.
The Detroit Review of Medicine and Pharmacy.
American Journal of Dental Science, Baltimore.
Archives of Dentistry.
L'art Dentaire, Paris.
Le Progres Dentare, Paris.
Deutsche Monatsschrift fuer Zahnheilkunde Leipzig.—Arthur Felix.
Ohio State Journal of Dental Science, Xenia, Ohio
Independent Practitioner, N. Y.
The Dental Record, London.
The Journal of the British Dental Association, London.
The Southern Dental Journal.
Items of Interest, Philadelphia.
The Dental Practitioner.—The Cincinnati "Medical" News.
The British Journal of Dental Science, London, Eng.
The Dental Advertiser.
The Messenger of Dentistry, Russian.
The Medical World.
The American Pharmacist.
The Nashville Journal of Medicine and Surgery.
Medical and Surgical Reporter, Philadelphia, Pa.
Dental Headlight.
Cincinnati Medical and Dental Journal.
The New York Medical and Surgical Journal.
The Dental Review.
The Western Dental Journal.

ESTABLISHED 1845.

### Ohio College of Dental Surgery.

Department of Dentistry—University of Cincinnati.

### SESSION 1888-89.

### FACULTY.

J. S. CASSIDY, M.D., D.D.S., Professor of Chemistry and Materia Medica.
H. A. SMITH, D.D.S., Dean of the Faculty, Professor of Operative Dentistry and Dental Pathology.
C. M. WRIGHT, D.D.S., Professor of Physiology and General Pathology.
WM. KNIGHT, M.D., D.D.S., Professor of Anatomy and Oral Surgery.
GRANT MOLLYNEAUX, D.D.S., Professor of Mechanical Dentistry and Metallurgy.

### DEMONSTRATORS.

B. C. HINKLEY, D.D.S., J. E. BARRICKLOW, D.D.S., } Demonstrators of Operative Dentistry.
G. S. JUNKERMAN, M.D., D.D.S., Demonstrator of Analytical Chemistry.
H. C. MATLACK, D.D.S., Demonstrator of Anatomy.
C. NEIDHAMER, Demonstrator of Mechanical Dentistry.

The Forty-Third Annual Winter Session begins Tuesday, October 2, 1888, and closes March 1, 1889.

The Annual Spring Session begins about April 1, 1889, and continues eight weeks.

The requirements for admission and graduation are those adopted by the National Association of Dental Faculties.

### FEES.

| | |
|---|---|
| Marticulation Fee (but once) | $ 5.00 |
| Professors' Tickets | 75.00 |
| Dissecting Ticket, including Material | 10.00 |
| Analytical Chemistry | 10.00 |
| Diploma Fee | 25.00 |
| Spring Course Ticket | 20.00 |

For further information and announcements, address

**H. A. SMITH, Dean, 128 Garfield Place, Cincinnati, Ohio.**

ESTABLISHED 1845.

### Ohio College of Dental Surgery.

Department of Dentistry—University of Cincinnati.

### SESSION 1888-89.

### FACULTY.

J. S. CASSIDY, M.D., D.D.S., Professor of Chemistry and Materia Medica.
H. A. SMITH, D.D.S., DEAN OF THE FACULTY, Professor of Operative Dentistry and Dental Pathology.
C. M. WRIGHT, D.D.S., Professor of Physiology and General Pathology.
WM. KNIGHT, M.D., D.D.S., Professor of Anatomy and Oral Surgery.
GRANT MOLLYNEAUX, D.D.S., Professor of Mechanical Dentistry and Metallurgy.

### DEMONSTRATORS.

B. C. HINKLEY, D.D.S., }
J. E. BARRICKLOW, D.D.S., } Demonstrators of Operative Dentistry.
G. S. JUNKERMAN, M.D., D.D.S., Demonstrator of Analytical Chemistry.
H. C. MATLACK, D.D.S., Demonstrator of Anatomy.
C. NEIDHAMER, Demonstrator of Mechanical Dentistry.

The Forty-Third Annual Winter Session begins Tuesday, October 2, 1888, and closes March 1, 1889.

The Annual Spring Session begins about April 1, 1889, and continues eight weeks.

The requirements for admission and graduation are those adopted by the National Association of Dental Faculties.

### FEES.

| | |
|---|---|
| Marticulation Fee (but once) | $ 5.00 |
| Professors' Tickets | 75.00 |
| Dissecting Ticket, including Material | 10.00 |
| Analytical Chemistry | 10.00 |
| Diploma Fee | 25.00 |
| Spring Course Ticket | 20.00 |

For further information and announcements, address

**H. A. SMITH, Dean, 128 Garfield Place, Cincinnati, Ohio.**

ESTABLISHED 1845.

### Ohio College of Dental Surgery.

Department of Dentistry—University of Cincinnati.

### SESSION 1888-89.

### FACULTY.

J. S. CASSIDY, M.D., D D.S., Professor of Chemistry and Materia Medica.

H. A. SMITH, D.D.S., Dean of the Faculty, Professor of Operative Dentistry and Dental Pathology.

C. M. WRIGHT, D.D.S., Professor of Physiology and General Pathology.

WM. KNIGHT, M.D., D.D.S., Professor of Anatomy and Oral Surgery.

GRANT MOLLYNEAUX, D.D.S., Professor of Mechanical Dentistry and Metallurgy.

### DEMONSTRATORS.

B. C. HINKLEY, D.D.S.,
J. E. BARRICKLOW, D.D.S., } Demonstrators of Operative Dentistry.

G. S. JUNKERMAN, M.D., D.D.S., Demonstrator of Analytical Chemistry.

H. C. MATLACK, D.D.S., Demonstrator of Anatomy.

C. NEIDHAMER, Demonstrator of Mechanical Dentistry.

The Forty-Third Annual Winter Session begins Tuesday, October 2, 1888, and closes March 1, 1889.

The Annual Spring Session begins about April 1, 1889, and continues eight weeks.

The requirements for admission and graduation are those adopted by the National Association of Dental Faculties.

### FEES.

| | |
|---|---|
| Marticulation Fee (but once) | $ 5.00 |
| Professors' Tickets | 75.00 |
| Dissecting Ticket, including Material | 10.00 |
| Analytical Chemistry | 10.00 |
| Diploma Fee | 25.00 |
| Spring Course Ticket | 20.00 |

For further information and announcements, address

H. A. SMITH, Dean, 128 Garfield Place, Cincinnati, Ohio.

ESTABLISHED 1845.

### Ohio College of Dental Surgery.

Department of Dentistry—University of Cincinnati.

---

### SESSION 1888-89.

---

### FACULTY.

J. S. CASSIDY, M.D., D D.S., Professor of Chemistry and Materia Medica.
H. A. SMITH, D.D.S., DEAN OF THE FACULTY, Professor of Operative Dentistry and Dental Pathology.
C. M. WRIGHT, D.D.S., Professor of Physiology and General Pathology.
WM. KNIGHT, M.D., D.D.S, Professor of Anatomy and Oral Surgery.
GRANT MOLLYNEAUX, D.D.S., Professor of Mechanical Dentistry and Metallurgy.

---

### DEMONSTRATORS.

A. O. ROSS, D.D.S., } Demonstrators of Operative Dentistry.
F. L. CARY, D.D.S., }
G. S. JUNKERMAN, M.D., D.D.S., Demonstrator of Analytical Chemistry.
H. C. MATLACK, D.D.S., Demonstrator of Anatomy.
G. W. GANDEE, D.D.S., Demonstrator of Mechanical Dentistry.

---

### The Forty-Fourth Annual Session

BEGINS TUESDAY, OCT. 1st, 1889.

---

### FEES.

| | |
|---|---|
| Marticulation Fee (but once) | $ 5.00 |
| Professors' Tickets | 75.00 |
| Dissecting Ticket, including Material | 10.00 |
| Analytical Chemistry | 10.00 |
| Diploma Fee | 25.00 |
| Spring Course Ticket | 20.00 |

For further information and announcements, address

**H. A. SMITH, Dean, 128 Garfield Place, Cincinnati, Ohio.**

### CONTENTS.

COMMUNICATIONS.

### THE DENTAL REGISTER,

A MONTHLY MAGAZINE DEVOTED TO THE INTERESTS OF THE

**DENTAL PROFESSION.**

*Terms Reduced to* $2.00 *Per Year. Single Copies,* 25 *Cents.*

**EDITED BY PROF. J. TAFT, D.D.S.**

—AND—

**Published by SAM'L A. CROCKER & CO., Ohio Dental and Surgical Depot.**

The volume commences in January and closes in December.

The REGISTER being issued monthly is always fresh, therefore Indispensable to the practitioner who desires to keep posted up with the new inventions and improvements which are daily developed. Neither expense nor effort will be spared to give value and variety to its contents. Articles will be illustrated with well executed engravings whenever they will add to the interest or assist to explanation.

Its extensive circulation and frequency of issue make it one of the BEST DENTAL ADVERTISING MEDIUMS in the United States.

All subscriptions must begin with January or July.

Local or special notices, five lines or less, will be inserted for $3 each insertion; over five lines, 50 cts. per line.

All advertising bills payable in advance, or at furthest, after the issue of the first number containing the advertisement. All transient advertisements to be paid for in advance.

☞CONTRIBUTIONS SOLICITED.

Exclusively Editorial Communications should be addressed to the Editor.

The latest number of the REGISTER may be ordered with or without other goods at any time, and all letters should be addressed to

**SAM'L A. CROCKER & CO., Ohio Dental and Surgical Depot, Cincinnati, O.**

